# Genomic Sequence of *Campylobacter jejuni* subsp. *jejuni* HS:19 Penner Serotype Reference Strain RM3420

**DOI:** 10.1128/genomeA.01701-16

**Published:** 2017-02-23

**Authors:** Craig T. Parker, Steven Huynh, Astrid P. Heikema

**Affiliations:** a Produce Safety and Microbiology Research Unit, Agricultural Research Service, U.S. Department of Agriculture, Albany, California, USA; b Department of Medical Microbiology and Infectious Diseases, Erasmus MC University Medical Centre, Rotterdam, The Netherlands

## Abstract

*Campylobacter jejuni* subsp. *jejuni* infections are a leading cause of foodborne gastroenteritis and the most prevalent antecedent to Guillain-Barré syndrome (GBS). Penner serotype HS:19 is among several capsular types shown to be markers for GBS. This study describes the genome of *C. jejuni* subsp. *jejuni* HS:19 Penner reference strain RM3420.

## GENOME ANNOUNCEMENT

Most *Campylobacter jejuni* subsp. *jejuni* infections result in an acute self-limited gastrointestinal illness (1). In a small number of cases, *C. jejuni* subsp. *jejuni* infections are followed by the development of the immune-mediated neuropathy, Guillain-Barré syndrome (GBS) (2). GBS can be elicited by *C. jejuni* sialylated lipooligosaccharides (LOS) that exhibit molecular similarity with gangliosides on human peripheral nerves (3–5). Several capsular types, including HS:19, are markers for GBS, suggesting that capsules located on the outer surface of *C. jejuni* may also contribute to GBS susceptibility (6). To further explore the role of capsular types in GBS, we report the genomic sequence of *C. jejuni* subsp. *jejuni* strain RM3420, the HS:19 Penner reference strain isolated from an enteritis patient at The Hospital for Sick Children, Toronto, Canada (7).

Genome sequencing was performed with Roche 454 FLX+ system with Titanium chemistry and Illumina MiSeq using shotgun library reads. A total of 223,770 454 reads with an average read length of 447 nucleotides (nt) were assembled *de novo* using the Roche Newbler assembler (version 2.3) and resulted in 53 total contigs (>100 bp). Reference assemblies against the *C. jejuni* NCTC 11168 (accession no. AL111168.1) and RM3196 (accession no. CP012690) were performed within Geneious software using Illumina reads (version 9.1). The *de novo* contigs and the contigs derived from the reference assembly were used to create a draft scaffold. The scaffold gaps were filled the perl script Contig_extender3 (8). The final sequence coverage was 122×. All base calls were validated and variations of the homopolymeric G tracts [poly(G) tracts] were characterized using the Illumina MiSeq reads within Geneious.

Protein-, rRNA- and tRNA-coding genes were identified using GLIMMER3 (9) within Geneious, RNAmmer (version 1.2) (10), and tRNAscan-SE (version 1.21) (11), respectively. The presence of bacteriophage-derived sequences was assessed using PHAST (12). The genomes were annotated based on those of the *C. jejuni* strains NCTC 11168 (GenBank accession no. AL111168) and RM3196 (GenBank accession no. CP012690). Additional annotations were performed using the identification of Pfam domains (version 26.0) (13) and BLASTP comparisons to proteins in the NCBI nonredundant database.

The complete annotated genomic sequence of RM3420 is 1.67 Mbp and contains 1,627 open reading frames. The RM3420 genome contains an additional 45 fragmented coding sequences (CDSs), identified as pseudogenes. Six flagellar modification genes and two capsular biosynthetic genes harbor poly(G) tracts; however, none of the poly(G) tracts within the flagellar modification genes display variability in length. RM3420 possesses the HS:19 capsular biosynthesis region (14) and a class A1 LOS biosynthesis locus (15) that includes a truncated version of the *cgtA* gene (annotated as a functional CDS, although it may be a pseudogene) and lacks poly(G) tracts. Similar occurrences of *cgtA* were found in the genomes of 2 GBS-related *C. jejuni* subsp. *jejuni* strains with the capsular serotype HS:41 and strain* C. jejuni* subsp. *jejuni* RM1285 (HS:19) (16,17). RM3420 also possesses a *C. jejuni* Mu-like prophage that carries the *dns* gene encoding an extracellular deoxyribonuclease, previously shown to inhibit natural transformation of *C. jejuni* (18).

### Accession number(s).

The whole-genome sequence and annotation were deposited with GenBank, BioProject, and BioSample under accession numbers CP017456, PRJNA206197, and SAMN05830826.
